# Knowledge, Attitudes, and Behaviours Concerning the Mediterranean Diet Among Older Adults in Australia

**DOI:** 10.1007/s10900-023-01237-1

**Published:** 2023-06-08

**Authors:** Ashlee Turner, Haley M LaMonica, Carissa Moroney, Fiona O’Leary, Sharon L Naismith, Victoria M Flood

**Affiliations:** 1 Faculty of Medicine and Health, Sydney School of Health Sciences, The University of Sydney, Sydney, NSW 2006 Australia; 2 Faculty of Medicine and Health, Translational Research Collective, The University of Sydney, Sydney, NSW 2006 Australia; 3 Charles Perkins Centre, The University of Sydney, Sydney, NSW 2006 Australia; 4 Faculty of Medicine and Health, Susan Wakil School of Nursing and Midwifery, Discipline of Nutrition and Dietetics, The University of Sydney, Sydney, NSW 2006 Australia; 5 Brain and Mind Centre, Healthy Brain Ageing Program, The University of Sydney, Sydney, NSW 2006 Australia; 6 Faculty of Science, School of Psychology, The University of Sydney, Sydney, NSW 2006 Australia; 7 Faculty of Medicine and Health, The University of Sydney, University Centre for Rural Health, Northern Rivers, NSW 2480 Australia

**Keywords:** Mediterranean diet, Knowledge, Attitudes, Behaviours, Older adults

## Abstract

Despite the growing body of evidence demonstrating the positive health effects of the Mediterranean diet, it is not routinely recommended in practice and adherence is low in the general population in Australia. The knowledge-attitude-behaviour model explains how health behaviours are supported through a process of acquiring knowledge, developing attitudes, and forming behaviours. Evidence has suggested that having a high level of nutrition-related knowledge is associated with more positive attitudes, which is directly linked to positive dietary behaviours. However, reports of knowledge and attitudes towards the Mediterranean diet, and how these directly relate to behaviours in older adults, are lacking. This study explored Mediterranean diet-related knowledge, attitudes, and behaviours among community-dwelling older adults in Australia. Participants were adults aged 55 years and older who completed an online survey that contained three parts: (a) knowledge – Mediterranean Diet Nutrition Knowledge Questionnaire (Med-NKQ); (b) nutrition-related attitudes and behaviours, and barriers and enablers to dietary change; (c) demographics. The sample included 61 adults who ranged in age from 55 to 89 years. The overall knowledge score was 30.5 out of a possible 40 points, with 60.7% classified as having a high level of knowledge. Knowledge was lowest for nutrient content and label reading. Attitudes and behaviours were generally positive and were not associated with level of knowledge. The most common barriers to dietary change were perceived cost and lack of knowledge, and motivational factors. There are a number of key gaps in knowledge that should be addressed through targeted educational programs. Strategies and tools to overcome perceived barriers and improve self-efficacy are needed to facilitate positive dietary behaviours.

**Supplementary Information:**

The online version contains supplementary material available at 10.1007/s10900-023-01237-1.

## Introduction

As the global population continues to grow, supporting the health and well-being of older adults is critical to ensure that they can continue to actively participate in society. Although there have been clear improvements in overall health along with increasing life expectancy, non-communicable chronic conditions continue to be the leading cause of disease burden and disability in older adults worldwide [1]. Poor diet quality is a major modifiable risk factor for many of these conditions, including cardiovascular disease (CVD) and type 2 diabetes [2], and contributes to approximately 5% of the total disease burden in Australia [3]. Specifically, diets low in legumes, wholegrains, fruits, and vegetables, and high in sodium, added sugar, alcohol, and red and processed meats, are responsible for much of this burden. Poor diet quality is common in older adults, with less than 8% meeting daily recommendations for fruit and vegetable intake [4]. Discretionary food intake is also high, accounting for approximately 33% of daily energy intake, with alcoholic beverages forming the largest source of energy from discretionary foods (approximately 7%) [5].

The Mediterranean diet is a dietary pattern that is rich in plant foods (e.g., wholegrains and cereals, fruits, vegetables, legumes, nuts, and seeds) with extra virgin olive oil as the primary source of fat, along with moderate intake of fish and seafood, poultry, eggs, dairy, and alcohol in the form of red wine; and low intake of red meat and processed foods high in saturated fat and sugar [6]. This pattern of eating is high in nutrients and dietary fibre, rich in antioxidant compounds and bioactive elements with anti-inflammatory properties, and is associated with nutritional adequacy in adult populations [7]. Because of these factors, there is extensive evidence to suggest a role for the Mediterranean diet in general healthy ageing, as well as in reducing CVD-related events, type 2 diabetes incidence, and overall mortality [2]. It has also been linked to benefits in cognition and dementia risk, and improvements in overall mood and symptoms of depression [8, 9]. Despite this growing body of evidence, the Mediterranean diet is not routinely used in practice for chronic disease management [10]. While adherence has been shown to be feasible and acceptable for adults in non-Mediterranean countries [11], adoption of the Mediterranean diet in the general population is low [12].

Behavioural change is complex, and a thorough understanding of the personal factors that underpin an individual’s dietary habits is vital when attempting to adopt a new dietary pattern. The knowledge-attitude-behaviour model explains how individual health behaviours follow a process of three stages: acquiring knowledge, developing attitudes or beliefs, and forming behaviours [13]. Alongside other influences of food choice, such as the broader social environment, food availability and affordability, taste preferences, and traditional cultural practices [14], knowledge and understanding can directly shape attitudes or beliefs, and together they influence behaviour. Indeed, studies have shown that knowledge of health-related behaviours is directly related to attitudes and practices in health management [15]. Nutrition knowledge covers a range of topics, including identifying food sources for particular nutrients, daily recommendations for food groups and nutrients, and understanding how diet is implicated in the maintenance of health and chronic diseases [16]. Overall, general nutrition knowledge tends to be poor among older adults, particularly compared to young and middle-aged adults [17, 18]. Having a high level of nutrition-related knowledge and more positive attitudes towards healthy eating is directly linked to positive dietary behaviours, including liking fruits and vegetables and proactively looking for nutrition information [19]. Although knowledge alone is not enough to produce behavioural change, it is critical for building the capability to change [20]. Assessing and analysing people’s nutrition-related knowledge, attitudes, and behaviours is a valuable method for gaining insight into the personal determinants of dietary intake and is an essential step for informing effective behaviour change interventions and evaluating nutrition education programs. Considering the role of diet, specifically the Mediterranean diet, in healthy ageing and chronic disease management, an in-depth understanding of older adults’ knowledge, attitudes, and behaviours in relation to the Mediterranean diet is needed. However, reports of knowledge and attitudes towards the Mediterranean diet and how these are directly related to behaviours are lacking. Thus, this study explored Mediterranean diet-related knowledge, attitudes, and behaviours among community-dwelling older adults in Australia.

## Methods

### Participants

A cross-sectional survey was conducted with 61 Australian adults aged ≥ 55 years. Participants were recruited via Twitter and the JoinUs Research Register, from October 2021 to June 2022, requesting voluntary participation. Qualtrics^XM^ survey software was used to construct and host the surveys. A link to the survey was disseminated via the recruitment methods described above, and included study requirements and possible risks for potential participants. Exclusion criteria included individuals < 55 years of age, non-residents of Australia, or those unable to complete the survey in English.

### Data Collection

Mediterranean diet knowledge, attitudes, and behaviours were assessed using a 41-item self-administered online questionnaire designed to be completed in approximately 15 minutes. No time restrictions were applied to complete the questionnaire, and the participants were required to answer every question in each part of the survey before proceeding to subsequent questions. The online questionnaire was divided into three parts:

Part A consisted of the knowledge component of the questionnaire, which was assessed using the Mediterranean Diet Nutrition Knowledge Questionnaire (Med-NKQ) [21]. The Med-NKQ is a 20-item tool that has been validated to assess knowledge of the Mediterranean diet in an Australian population [21]. The questionnaire assessed three major domains: (a) dietary patterns, foods, and nutrients to reduce cardiac risk factors (four questions); (b) nutrient content of foods, quantification, and food label reading (seven questions); and (c) core Mediterranean diet foods, dietary patterns, and meal selection (nine questions). Scores were calculated for overall knowledge and for each of the three domains, with higher scores indicating greater knowledge (see Table 1 for a summary of scoring). The Med-NKQ is shown in Supplementary Material 1.

**Table 1:** Summary of the Med-NKQ Scores (n = 61)

Domains	Related questions	Score range	Mean score	SD	Median	IQR
CVD risk factors	1, 2, 4, 5	0–10	8.4	1.6	9	8–9
Nutrient content of foods, quantification, and label reading	3, 6, 7, 8, 9, 19, 20	0–15	10.0	2.8	10	9–12
Core MD foods, patterns, and meal selection	10, 11, 12, 13, 14, 15, 16, 17, 18	0–15	12.2	2.4	13	10.5–14
Total		0–40	30.5	5.0	31	28–34

Abbreviations: CVD, cardiovascular disease; MD, Mediterranean diet; SD, standard deviation; IQR, interquartile range

Part B assessed participants’ attitudes and behaviours towards the Mediterranean diet, as well as barriers and facilitators to changing their habitual diet. Attitudes were assessed via three statements related to everyday diet and food choices and scored on a 7-point Likert scale (response options ranging from ‘strongly disagree’ to ‘strongly agree’) [22]. These questions have been previously used to measure nutrition-related attitudes in older adults [22]. The three statements were as follows: (1) The healthiness of food has little impact on my food choices; (2) It is important for me that my daily diet contains a lot of vitamins and minerals; and (3) I eat what I like and do not worry much about the healthiness of food. Two of the three statements had an inverse response scale and were recoded accordingly. The total attitude score was calculated by summing the three items (ranging from to 3–21 with higher scores indicating a more positive attitude towards healthy foods and diet). Behaviours were assessed using four questions. The questions were developed and adapted from previous surveys [19, 23, 24]. Part B of the survey is provided in Supplementary Material 2.

Part C consisted of open- and closed-ended questions related to the participants’ demographic characteristics, including age, sex, level of education, country of birth, language spoken at home, diagnosed health conditions, height, and weight.

### Data Analysis

Data were analysed using Statistical Package for the Social Sciences (SPSS, Version 28). Descriptive data are expressed as means ± standard deviations for continuous data and as frequencies and percentages for categorical data. Total and individual domain knowledge scores were dichotomised as high knowledge and low knowledge, and the total attitude score was dichotomised as positive and negative using the median split method [25]. This choice was made because of the (1) asymmetrical distribution of the raw scores and (2) infrequent occurrence of extremely low values. Behaviours were dichotomised as positive or negative. Associations between nutritional knowledge, attitudes, and sociodemographic variables were examined using non-parametric and Fisher’s exact tests. A *p*-value of < 0.05 was used to determine significance (two-tailed).

## Results

### Participant Characteristics

Of the 92 participants who agreed to complete the online survey, 31 either did not answer all the questions or did not complete the survey to the end. Therefore, 61 participants provided valid responses and were included in the analysis. The median time to complete the survey was 12 minutes (interquartile range [IQR]: 8.7–15.5).

A summary of participants’ demographic characteristics is presented in Table 2. Participants ranged in age from to 55–89 years (63.7 ± 8.1). Among the participants, 73.8% (*n = *45) were female, 70.5% (*n = *43) had completed some form of tertiary-level education, 67.2% (*n =* 47) were born in Australia, and 75.4% (*n =* 46) lived in metropolitan areas.

**Table 2:** *Demographic Characteristics of Study Participants (n = 61)*

*Continuous variables*	Mean	SD
Age, years	63.7	8.1
Body mass index, kg/m^2^	25.4	5.0
*Categorical variables*	n	%
Sex		
- Male	16	26.2
- Female	45	73.8
Education level		
- Postgraduate	31	50.8
- Bachelors	12	19.7
- Advanced certificate/diploma	15	24.6
- Secondary (Year 7–12)	3	4.9
Country of birth		
- Australia	41	67.2
- United Kingdom	12	19.7
- New Zealand	1	1.6
- Other*	7	11.5
Language spoken at home		
- English only	57	93.4
- Other†	4	6.6
State		
- New South Wales	39	63.9
- Queensland	10	16.4
- Victoria	4	6.6
- South Australia	4	6.6
- Western Australia	3	4.9
Diagnosed health condition, % yes	41	67.2
- Cancer	6	9.8
- Diabetes	3	4.9
- Heart disease	8	13.1
- Hypertension	4	6.6
- Kidney disease	1	1.6
- Lung condition	2	3.3
- Mental health condition	11	18.0
- Stroke	2	3.3
- Other‡	19	31.1
Main grocery shopper		
- Yes	34	55.7
- No	4	6.6
- I share the responsibility	23	37.7

Abbreviations: SD, standard deviation

* Includes India, the USA, Hong Kong, Papua New Guinea, and Nigeria. † Includes Cantonese, French, Danish, and Estonian. ‡ Includes hypercholesterolemia, osteoarthritis, Hashimoto’s disease, diverticulitis, fibromyalgia, and osteoporosis

### Mediterranean Diet Knowledge

As shown in Table 1, out of a possible 40 points on the Med-NKQ, the mean ± standard deviation (SD) and median score (IQR) for the whole sample was 30.5 ± 5.0 and 31 (IQR: 28–34), respectively. The total scores ranged from 17 to 40. More than half of the participants had high total knowledge (*n =* 37, 60.7%), whereas almost 40% had low overall knowledge (*n =* 24, 39.3%). Sex, level of education (tertiary vs. non-tertiary), and area classification (metropolitan vs. rural) did not differ between the two groups (all *p* > 0.05). There was also no difference between the groups in terms of age (*U =* 429.50, *p =* 0.830) or body mass index (BMI) (*U =* 335.00, *p =* 0.190).

#### Domain 1 – Dietary Patterns, Foods, and Nutrients for Reducing Cardiac Risk Factors

Of the possible 10 points, the mean ± SD and median (IQR) scores were 8.4 ± 1.6 and 9 (IQR: 8–9), respectively. Scores ranged from 1 to 10. Thirty-six participants were classified as having high knowledge (59.0%) and 25 were classified as having low knowledge (41.0%). There were no differences between the groups in terms of sex, level of education, area classification, age, or BMI (all *p* > 0.05).

Thirteen participants (21.3%) scored full points in this domain. There were two questions within this domain where 75% or more of the participants scored full points (questions 4 and 5). These questions covered general nutritional facts and foods that increase blood cholesterol levels. On a question asking for correct statements about foods for lowering blood cholesterol, less than 50% of respondents scored full points (question 1), with 21 participants (34.4%) incorrectly identifying reducing salt consumption as a strategy to reduce cholesterol.

#### Domain 2 – Nutrient Content of Foods, Quantification, and Food Label Reading

Out of a possible 15 points, the mean ± SD and median (IQR) scores were 10.0 ± 2.8 and 10 (IQR: 9–12), respectively. Scores ranged from 2 to 15. Thirty-nine participants (63.9%) had higher knowledge than 22 (36.1%) with low knowledge. Those who had a high level of knowledge in this domain were more likely to be younger (*mean age =* 62.05, *SD =* 7.59) than those classified as having low knowledge (*mean age =* 66.68, *SD =* 8.33, *U =* 277.50, *p =* 0.023). There were no differences between the groups in terms of sex, level of education, area classification, or BMI (all *p* > 0.05).

Only two participants (3.3%) scored full points in this domain. There were two questions within this domain where 75% or more of the respondents scored full points (Questions 7 and 19). These questions covered foods with good sources of fibre and the ability to accurately read nutrition information panels to identify the product with the highest amount of salt. There were two questions in which less than 50% of the sample scored full points (questions 8 and 9), both of which were pictorial. Question 8 asked participants to correctly identify foods that were high in salt. Although 59 respondents (96.7%) correctly identified deli ham as being high in salt, more than half (n = 32, 54.2%) were unable to correctly identify smoked salmon as being high in salt, resulting in a loss of points. Question 9 asked participants to select the alcoholic beverage with the lowest number of standard drinks (200mL red wine vs. 375mL full-strength beer vs. 60mL dark spirit); more than half of the participants (57.4%) were unable to do so. Regarding the questions related to label reading of packaged foods, 85.2% of participants were able to correctly identify the product with the highest amount of salt. However, only 54.1% of the participants were able to correctly identify the product that was better for heart health based on the nutrition information panel.

#### Domain 3 – Core Mediterranean Diet Foods, Dietary Patterns, and Meal Selection

Of the possible 15 points, the mean ± standard deviation and median (IQR) scores were 12.2 ± 2.4 and 13 (IQR: 10.5–14), respectively. The scores ranged from 4 to 15. Thirty-one participants were classified as having high knowledge (50.8%) and 30 as having low knowledge (49.2%). Those classified as having high knowledge in this domain were more likely to have a significantly lower BMI (*mean BMI =* 23.33, *SD =* 5.43) than those with a low level of knowledge (*mean BMI =* 27.33, SD = 3.55, *U =* 238.00, *p =* 0.003). The groups did not differ in terms of sex, education level, area classification, or age (all *p* > 0.05).

Ten participants (16.4%) scored full points in this domain. There were three questions within this domain where 75% or more of the participants scored full points (questions 10, 13, and 18). These questions covered meal options that aligned with the Mediterranean diet and foods used to add flavor to meals. Less than 50% of participants were able to correctly identify cooking methods typical of the Mediterranean diet. Although 56 participants (91.8%) identified moist cooking methods, such as stewing, as the most typical method, more than half (n = 29, 51.8%) of those lost points due to incorrect identification of additional cooking methods.

### Attitudes and Behaviours

When asked about their current diet, 38 respondents (62.3%) rated their quality as excellent or very good and were classified as having a high-quality diet, and 23 participants (37.7%) rated their diet of average or poor quality and were classified as having a low-quality diet. Notably, the probability of subjectively rating diet as high quality was higher among those with a low level of overall knowledge than among those with a high level of knowledge (*p =* 0.034).

**Fig. 1:**
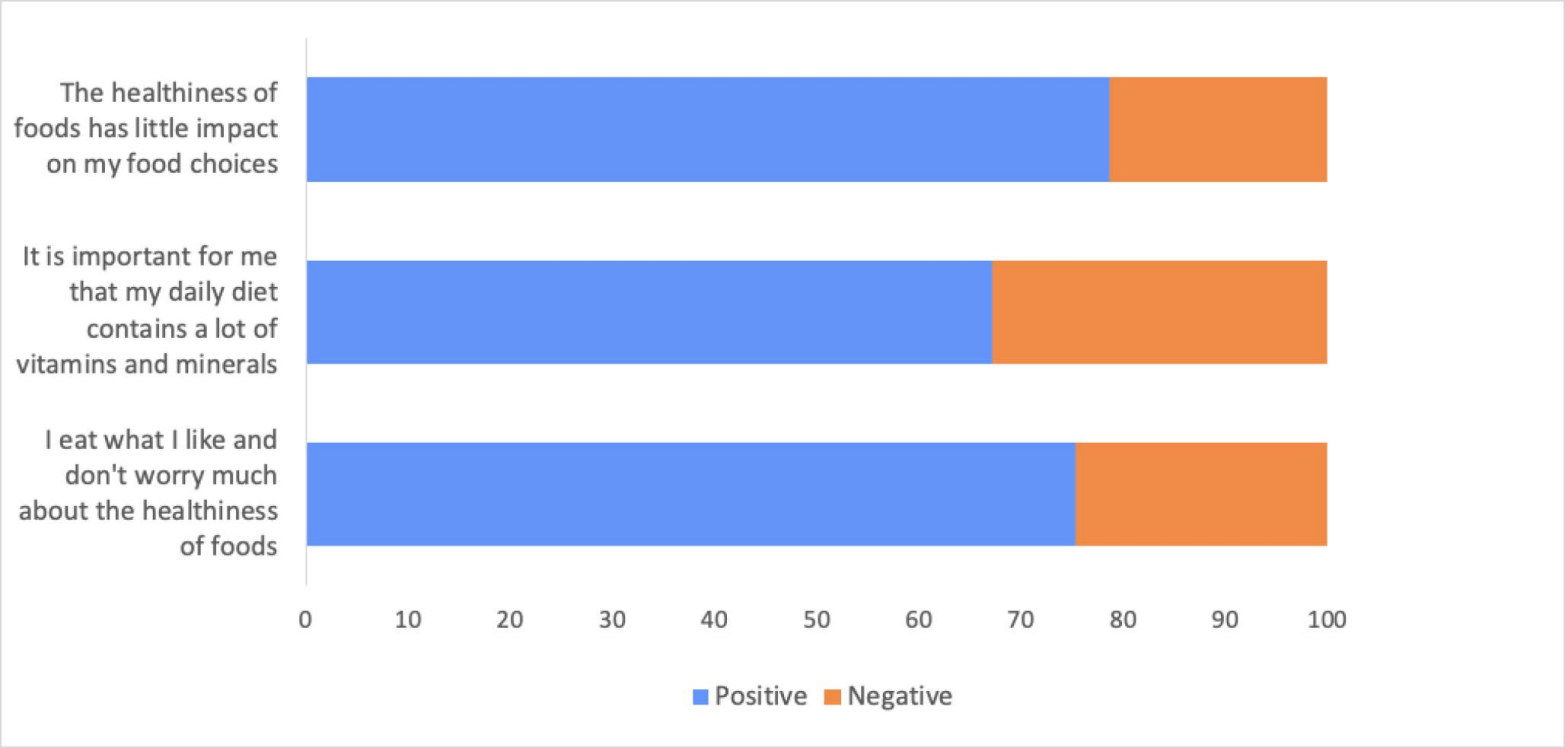
Nutrition-Related Attitudes (n = 61)

Fig. 1 shows the attitudes of older adults towards nutrition. Just over half of the sample (*n =* 34, 55.7%) were classified as having an overall positive attitude towards food choices and healthy eating. There were no differences in age, BMI, sex, level of education, or area classification between those with an overall positive attitude and those with a negative attitude (all *p* > 0.05). There was also no difference in attitudes between those with a high level of knowledge and those with a low level of knowledge (all *p* > 0.05). However, those with an overall positive attitude towards food choices and healthy eating were more likely to subjectively rate their diet quality as high, whereas those with a positive attitude were four times more likely to rate their diet as good quality compared to those with a negative attitude (*OR =* 4.06, 95%CI [1.3559 to 12.1722], *p =* 0.01).

Nutrition-related behaviours were overwhelmingly positive, with 53 participants (86.9%) stating that they proactively sought out nutrition information and 59 (96.7%) stating that they read the nutrition information panel sometimes or most of the time. In terms of specific eating behaviours, 48 participants (78.7%) stated that they were interested in tasting new foods that they had not tasted before, and 50 (82.0%) preferred to eat home-cooked meals rather than eating out or ready-made meals.

### Barriers and Enablers to Adopting a New Dietary Pattern

#### Barriers

**Fig. 2:**
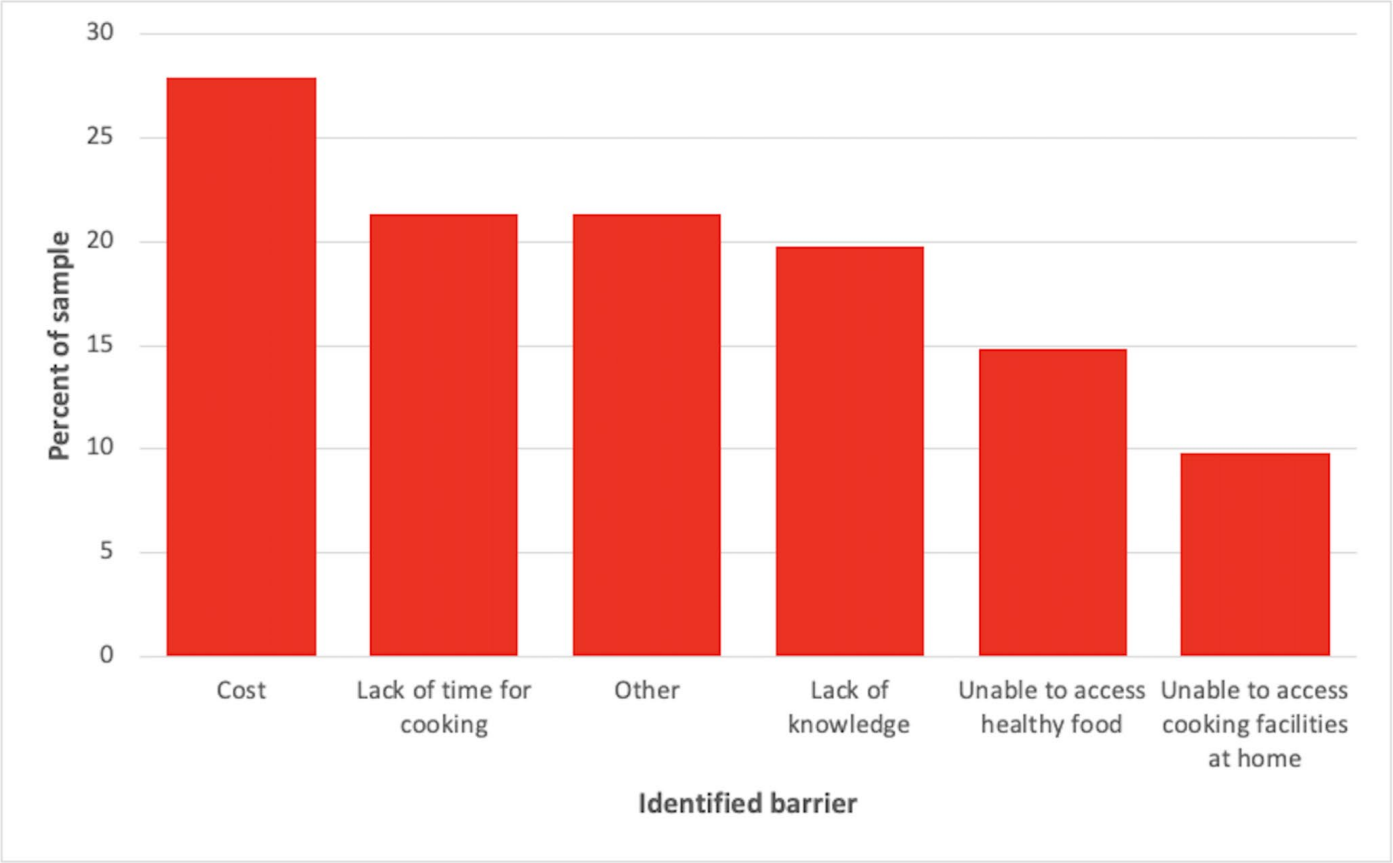
Barriers to Adopting a New Dietary Pattern (n = 61)

The percentage of participants who endorsed a particular barrier is shown in Fig. 2. Almost one-third of the participants (27.9%) stated that the cost was a deterrent to trying a new diet. 20% of the sample stated that a lack of knowledge and time for cooking would make it more difficult to adopt a new dietary pattern, whereas a third of participants (34.4%) identified that taste preferences would make it harder. Other barriers (entered as free text by respondents) to changing habitual diets included living with others who have different food preferences and not wanting to cook more than one type of meal, food intolerance, and a lack of interest in cooking. There were no differences in the number or type of barriers identified between those with high and low levels of total and individual domain knowledge (all *p* > 0.05).

#### Enablers

**Fig. 3:**
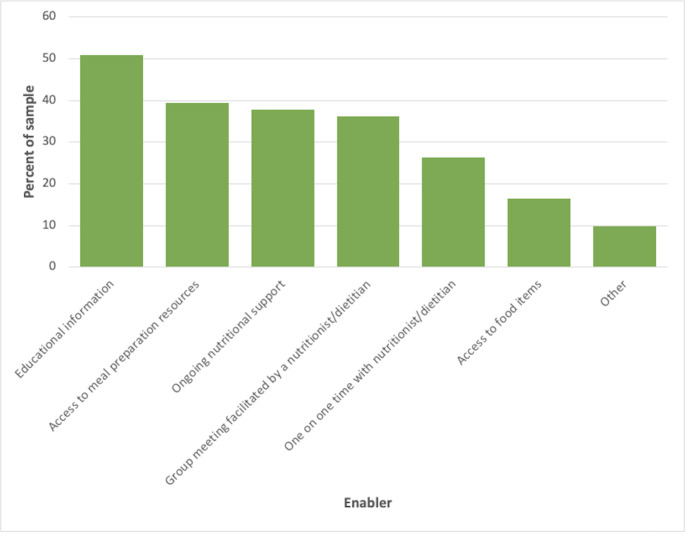
Enablers to Adopting a New Dietary Pattern (n = 61)

The percentage of participants who endorsed a particular enabler is shown in Fig. 3. 50% of participants reported that educational information would help them with dietary changes. Approximately 40% of the participants stated that ongoing nutritional support and access to meal preparation resources, such as meal plans and recipes, would make them more willing to make changes to their current diet. Other enablers to adopting a new dietary pattern included clear evidence of effectiveness and more ‘yumminess’ factor.

## Discussion

This study provides an overview of older Australian adults’ Mediterranean diet knowledge and attitudes as well as perceived enablers and barriers to changing their habitual diet. This study aimed to understand how much older adults in a non-Mediterranean community know about the Mediterranean diet and how this relates to nutrition-related attitudes and behaviours. Most participants in our sample were female, with a high level of education, and English as their first language. The main findings of this study are as follows: (1) more than half of our sample had a high level of Mediterranean diet knowledge; (2) nutrition-related attitudes were generally positive, irrespective of knowledge level; and (3) there were several perceived barriers identified, including cost and family food preferences, that impede older adults ability to make changes to their habitual diet.

The findings from this study indicate that even in this highly educated and generally well-informed sample, there are still some key area gaps in knowledge, and any strategy to address these are likely to be beneficial for the entire population. These findings are consistent with those obtained from a sample of Australian cardiac patients [21]. Indeed, across multiple Australian populations, there is a lack of understanding about the nutrient content of foods that are not commonly consumed in the standard Australian diet (e.g., smoked salmon) [26], alcoholic content of beverages, and cooking methods typical of the Mediterranean diet. Conversely, knowledge of what foods provide a good source of fibre and foods that raise blood cholesterol, as well as the ability to identify certain nutrients from a nutrition information panel and meal options that align with the Mediterranean diet are generally strong [21]. This highlights that there are consistent strengths and weaknesses in Mediterranean diet knowledge across Australian populations, and provides a strong case for specific education programs that target the known gaps.

Interestingly, we found that overall knowledge did not seem to differ between males and females, or between those with varying levels of education. This contrasts with many previous studies that have demonstrated that females and those with higher levels of education tend to have greater nutritional knowledge [27]. The contrasting findings are likely related to the small and relatively homogenous sample size, and caution should be exercised when generalising beyond this sample. The results are still valuable in highlighting key knowledge deficits that are present across multiple Australian populations to which education programs can be targeted. Given the demographic bias of the sample, the real picture in Australia is likely to be worse than suggested by these results. Collecting nationally representative data would be useful in developing national education programs tailored to the needs of a broader population.

CVD continues to be the leading cause of death in Australia, and poor diet contributes to approximately 50% of the disease burden [28]. In this sample, more than half of the participants reported having at least one chronic condition, and one-quarter of the sample reported having a condition for which dietary risk factors contribute to a significant proportion of the disease burden [3], highlighting the need to maintain and improve health through diet. Although overall knowledge of dietary patterns, foods, and nutrients for reducing cardiac risk is generally sound, there are a range of individual areas where knowledge is suboptimal and represents a target for improving knowledge, particularly in the context of cardiovascular health. For example, although participants were able to correctly identify packaged foods high in salt based on the nutrition information panel, less than 50% of the sample were able to correctly identify foods for lowering blood cholesterol and foods high in salt. Given the role of cholesterol and sodium in CVD and hypertension, both of which are common conditions associated with ageing [28], these knowledge gaps are significant. Additionally, much of the sample had a poor understanding of the alcoholic content of the beverages. This is in keeping with previous studies that have illustrated that older adults tend to have poor knowledge of safe drinking guidelines and the risks associated with their own alcohol consumption [29]. Heavy alcohol consumption is a well-known contributor to the CVD disease burden and a range of other serious health effects [30]. Alcohol consumption is high among older adults, and the proportion of those aged 50 years and older who drink at risky levels has increased substantially in Australia [31]. Recent modelling evidence has suggested that even a slight reduction in alcohol intake, equivalent to approximately three-quarters of a standard drink, would be beneficial for reducing overall energy intake and the contribution of discretionary foods to daily energy intake [32]. This finding has implications for public education campaigns and highlights the need for clear information and targeted approaches to address and improve population health.

There is evidence that the public is generally aware of dietary guidelines, which has increased over time [33]. Indeed, approximately 65% of the sample were able to correctly identify the daily recommended number of servings of vegetables, which is similar to findings from other Australian studies [33]. However, there is an obvious gap between relevant knowledge and actual eating behaviours, given that adherence to dietary guidelines in Australia is low across the population [34]. Awareness of dietary intake is also a key determinant of dietary quality. In this study, those who had a low level of overall knowledge were more likely to subjectively rate their current diet as high-quality compared to those with a high level of knowledge. However, those who are more knowledgeable are likely to be better equipped with an understanding of what constitutes a high-quality diet. Given the generally poor diet quality of older adults in Australia, it is possible that there is a disconnect between individual perceptions of diet quality and actual diet quality in this group. These findings are consistent with the Dunning-Kruger effect, a type of cognitive bias in which individuals overestimate their abilities, often because those with lower levels of knowledge have not developed the necessary understanding and skills to accurately evaluate their own health knowledge [35]. Data from the United States suggest that inaccurate identification of diet quality is common, with a large percentage of the population perceiving their diet to be of higher quality than indicated by objective measurements [36]. Older adults have also been shown to overestimate adherence to vegetable intake [37]. Overestimation of diet quality is problematic and highlights a group who may be at greater risk of chronic disease because those who misperceive their diet quality as high are unlikely to see a need to change their diet and therefore may not respond to health promotion messages. Thus, strategies are needed to address this cognitive bias and to overcome this barrier. Although we did not collect dietary intake data and we are unable to directly comment on whether misperceptions of diet quality versus actual diet intake were present in this sample, it is an important consideration in the context of health promotion and an area for future research.

Our results provide new insights into the factors that influence an individual’s intention to change their diet. Although it has been shown that older adults in Australia can adhere to a Mediterranean diet in short-term intensive interventions [38], the reality of widespread dietary behaviour change at the population level, where differences in food habits, preferences, and cultural influences are extensive, is more complex. Participants acknowledged several economic and practical barriers that would impact their capacity for dietary change, including cost, knowledge, and time. Cost and affordability were the most common barriers, despite the sample being highly educated and likely from a higher-income bracket. This is similar to findings from other studies that have reported that increasing food costs and a lack of affordability are important barriers to general healthy eating [39] and adherence to the Mediterranean diet among adult populations in non-Mediterranean regions [40]. Evidence preceding the COVID-19 pandemic suggests that the Mediterranean diet may be more cost-effective than the typical diet of Australians [41]. With the increasing cost of living, the cost of eating a healthy diet – particularly one that includes fresh fruits, vegetables, and whole grains – has increased markedly due to supply chain disruptions and global inflation as a result of the COVID-19 pandemic. However, the overall food costs associated with consuming a healthy diet continue to be lower than those of the standard habitual diet [42]. There is also evidence to suggest comparable nutrient content between fresh and frozen or canned fruits and vegetables, which are more accessible and tend to come at a cheaper price point [43]. Thus, cost may only be a perceived barrier, and education could potentially address this concern. The provision of educational information and meal preparation resources were the most cited enablers of changing the habitual diet in this sample. Findings from an Australian study suggest that acceptance of the Mediterranean diet can be improved by providing simple resources, including educational material, advice for recipe modification, clear recommendations for daily and weekly servings of core food groups, and templates for creating individual shopping lists and meal plans [40]. However, nutrition professionals have identified a lack of patient education resources specific to the Mediterranean diet, particularly those that provide practical advice that is simple and culturally appropriate [10]. It is clear that developing targeted educational information, including how to create a cost-effective and nutritionally balanced diet (for example, purchasing home-brand products, buying in-season or frozen fruits and vegetables, and swapping meat for legumes as an alternative protein source) is needed.

Motivation is also a key component of health behaviour change, directly impacting the intention to change behaviour [20]. In the present study, participants indicated that the individual food preferences of other household members and a lack of interest in cooking were key barriers that influenced their motivation to change their habitual diet. Consistent with this, family food preferences are a key barrier to healthy eating [44] and adapting to a Mediterranean diet in non-Mediterranean countries [45]. Social support is a key determinant of dietary adherence and can act as an intermediary to stressors that are often experienced when making lifestyle changes [44]. In addition, cooking and food preparation are key activities for maintaining independent living, and enjoyment of cooking is a key element of cooking skills, both of which play a direct role in adopting healthy eating patterns and meeting dietary guidelines [46]. Importantly, evidence suggests that dietary behaviour change is guided by willpower and willingness to change [47], highlighting the importance of improving self-efficacy. Self-efficacy is a key determinant of positive outcomes in lifestyle interventions and is integral for facilitating and improving adherence to a Mediterranean diet [48]. However, commentary on the self-efficacy of older adults in Australia to adapt their eating habits in the context of perceived barriers is scarce. Strategies to support and improve self-efficacy and intrinsic motivation are likely to be beneficial for providing older adults with tools to overcome perceived barriers and make positive dietary changes.

Our study had several limitations that should be considered. First, the population was a small, relatively homogenous convenience sample of older adults who were mostly aged 65 years or younger, well educated, and had English as their first language, which may not be representative of the general older Australian population. Given that more than 30% of the older adult population in Australia has a culturally and linguistically diverse (CALD) background [28], which has not been captured in this sample, future studies would benefit from investigating Mediterranean diet knowledge across different CALD subgroups to design inclusive and appropriate interventions for the general population. The use of social media platforms as part of our recruitment strategy may have resulted in selection bias given that approximately 65% of the cohort recruited were < 65 years old (i.e., those older than 65-years may be less likely to use social media platforms). An online survey was selected to increase the reach and response rate; however, we acknowledge that not offering a paper-based alternative to the online option may have produced biased results by excluding a subgroup of potential participants who lacked access to the Internet or digital literacy to complete an online survey [49]. Additionally, response rates to online surveys are lower than those for face-to-face or paper-based surveys, and participants are more likely to exit before completing the survey [50]. To overcome this, the survey was designed to take 15 minutes to complete, and we limited the use of the open-ended questions. Importantly, we did not collect dietary intake data; therefore, we cannot draw conclusions about the links between nutrition-related knowledge, attitudes, behaviours, and eating habits in this population. Finally, while the Med-NKQ has been tested for repeatability in a cardiac population of similar age demographics [21], it is yet to be implemented in an intervention study to measure and understand changes following education, and there is no indication of how much knowledge is needed to impact healthy eating. Future research would benefit from investigating the utility of the Med-NKQ to measure changes following dietary education and to identify if there is a critical cut-off point of knowledge to incite dietary behaviour change.

## Conclusions

To our knowledge, this is the first Australian study to assess the knowledge, attitudes, and behaviours of older adults towards the Mediterranean diet. The findings indicate that even in this highly educated and generally well-informed sample, a few key knowledge deficits appear to be consistent across multiple cohorts in Australia, highlighting the need for targeted education programs. The study also highlighted several potential strategies and tools to support dietary change by addressing perceived barriers, including the provision of simple and practical resources, detailing how to construct a cost-effective and nutritionally balanced diet. Despite the importance of motivation and self-efficacy, there has been little consideration of these factors in intervention design, and they are likely to be critical targets for future research in Australia. Finally, examining the association between Mediterranean diet knowledge and adherence in Australian older adults and how strategies to overcome perceived barriers and improve self-efficacy impact dietary change and health outcomes warrant further exploration.

### Electronic Supplementary Material

Below is the link to the electronic supplementary material.

Supplementary Material 1

Supplementary Material 2

## Data Availability

The data that support the findings of this study are available from the corresponding author upon request.
