# Supplementary material for: Knowledge, Attitudes, and Behaviours Concerning the Mediterranean Diet Among Older Adults in Australia
Source: J Community Health. 2023 Jun 8;48(6):951–62. doi: 10.1007/s10900-023-01237-1 (PMC10248335; doi:10.1007/s10900-023-01237-1)
Supplement: Supplementary file 1 — Supplementary Material 1 [file 10900_2023_1237_MOESM1_ESM.pdf]

1. Which of the following statements is true if you want to lower your blood cholesterol? You may choose more than one.

☐ Eat more fruits and vegetables

☐ Eat more wholegrain foods

☐ Eat less fatty and processed meats

☐ Eat more dairy products

☐ Eat less salt on your food

2. Which of the following statements are true if you want to lower your blood pressure? You may choose more than one.

☐ If using packaged foods, choose products with less salt

☐ If you are overweight, try to lose some weight

☐ Drink more alcohol

☐ Eat more dark leafy greens

☐ Eat more white bread

3. What is the recommended number of servings of vegetables per day? 1 serve = ½ cup cooked vegetables **or** 1 cup raw vegetables/salad.

☐ 2 serves

☐ 3 serves

☐ 5 serves

☐ 7 serves

4. Which of the following statements are true? You may choose more than one.

☐ The only nutritional benefit of fruits and vegetables is vitamins and minerals

☐ Fat is always bad for your health; you should therefore avoid it as much as possible

☐ Dietary fibre is important for lowering your cholesterol

5. Which of the following foods are more likely to raise your blood cholesterol? You may circle more than one.

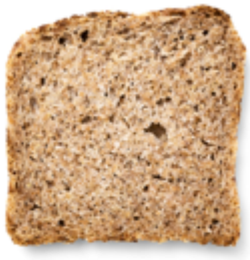

Wholemeal bread

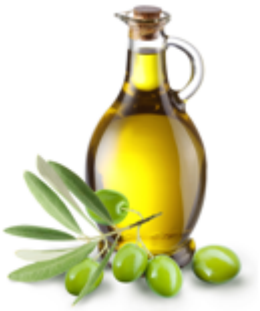

Extra Virgin olive oil

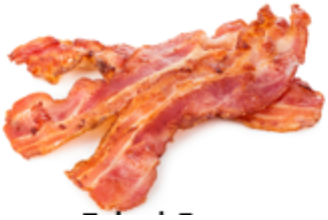

Fried bacon

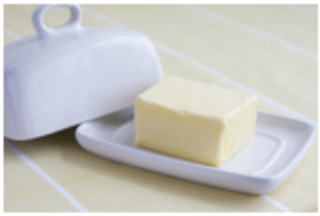

Butter

6. Circle the foods that are a source of healthy fats. You may circle more than one.

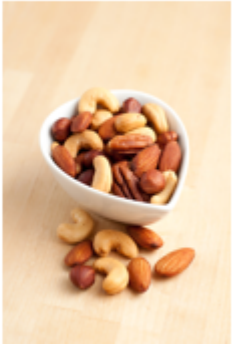

Unsalted nuts

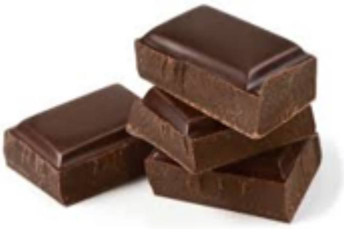

Dark chocolate

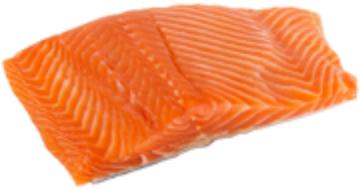

Salmon fillet

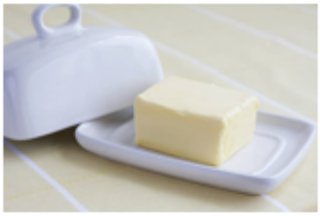

Butter

7. Which of the following foods are a good source of fibre? You may circle more than one.

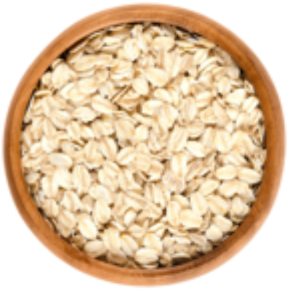

Rolled oats

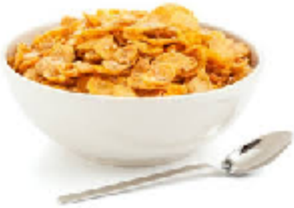

Corn flakes

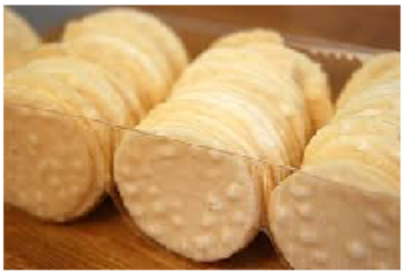
Rice crackers

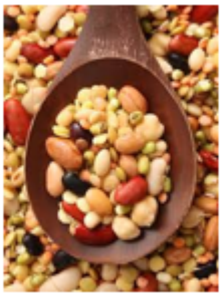
Legumes

8. Which of the following foods are high in salt? You may circle more than one.

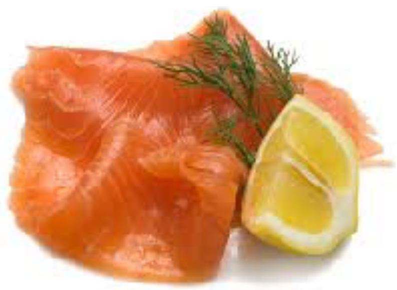
Smoked salmon

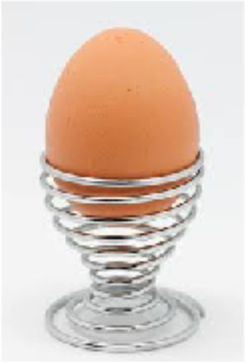
Boiled egg

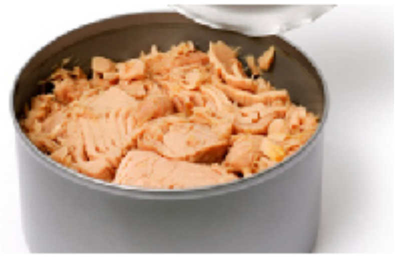
Canned tuna in spring water

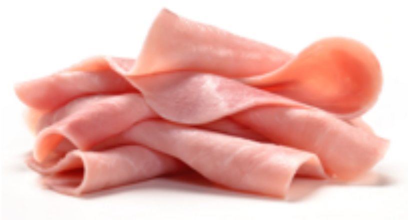
Deli ham

9. Which alcoholic beverage below contains the lowest number of standard drinks?

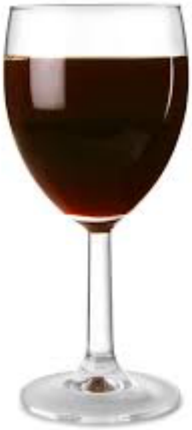

200mL red wine

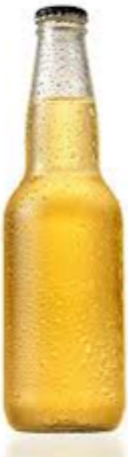

375mL full strength beer

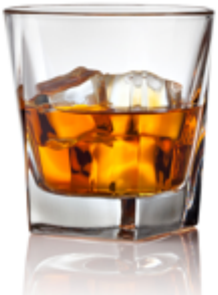

60mL of dark spirit

10. Which pasta meal better reflects the Mediterranean diet principles for heart health? Circle the correct response.

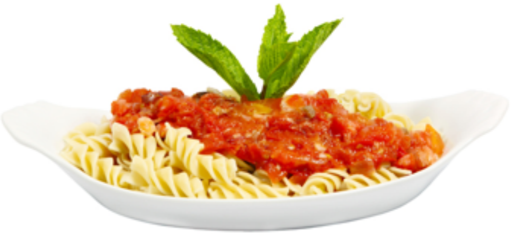

Pasta with tomato sauce

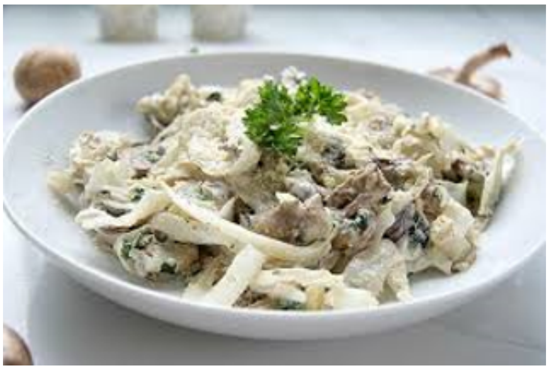

Pasta with mushroom and cream sauce

11. Which of the following foods are considered core components of the Mediterranean diet? You may circle more than one.

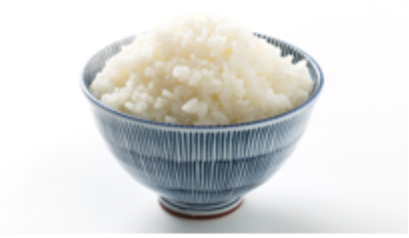

White rice

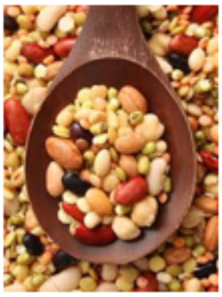

Legumes

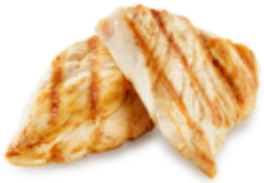

Chicken breast

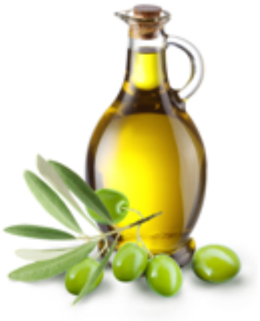

Extra Virgin olive oil

12. Which of the following foods are considered core components of the Mediterranean diet? You may circle more than one.

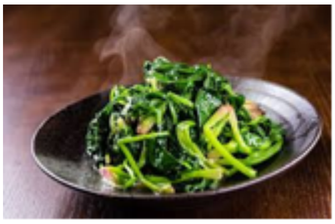

Leafy greens

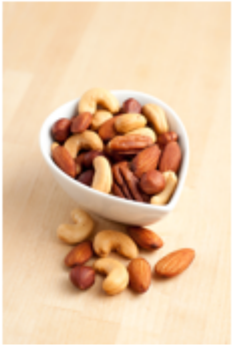

Unsalted nuts

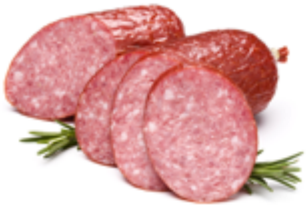

Salami

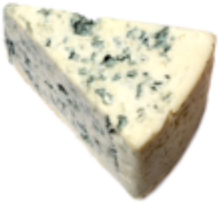

Blue cheese

13. Which of the below are used in the Mediterranean diet to add flavour to food? You may circle more than one.

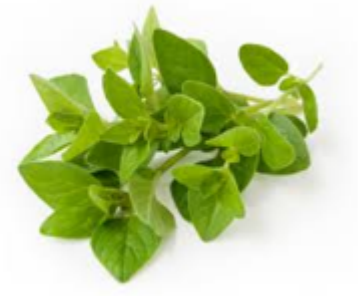

Oregano

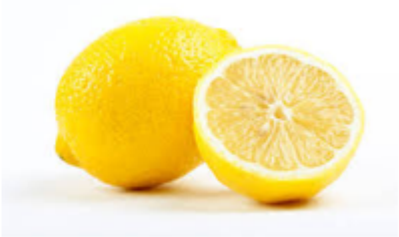

Lemon juice

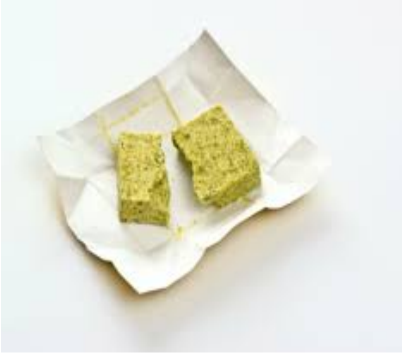

Powdered stock cube

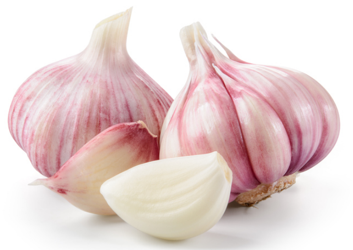

Garlic

14. Which of the following cooking methods is most typical of the Mediterranean diet? You may choose more than one answer.

☐ High heat stir-fry
☐ Use of moist cooking methods such as stewing
☐ Barbeque
☐ Only eating raw foods

15. Which of the following statements is true about the Mediterranean diet? You may choose more than one answer.

☐ Use of Extra Virgin Olive Oil as main oil
☐ Fruit juice consumed with all meals
☐ Eating red meat 3-4 times per week
☐ Mostly plant-based diet

16. Which of the following drinks is consumed the most as part of the Mediterranean diet?

☐ Water
☐ Fruit juice
☐ Wine
☐ Coffee

17. Which of the following meals would be considered the best choice if following the Mediterranean diet for heart health?

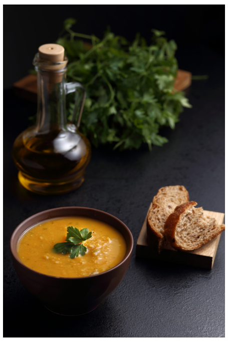

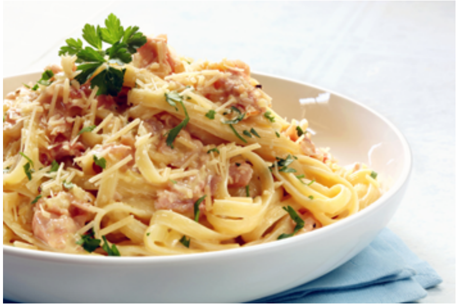

Vegetable and lentil soup with wholegrain bread and olive oil

Carbonara

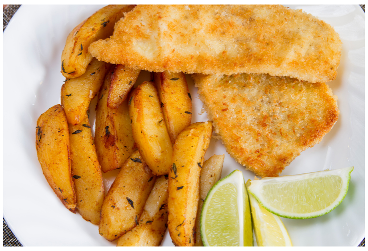

Oven baked crumbed fish and potato

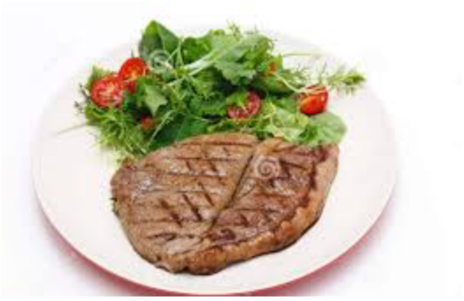

Grilled steak and garden salad with fat free dressing

18. If you were having breakfast in a café, which menu option would best fit the Mediterranean diet?

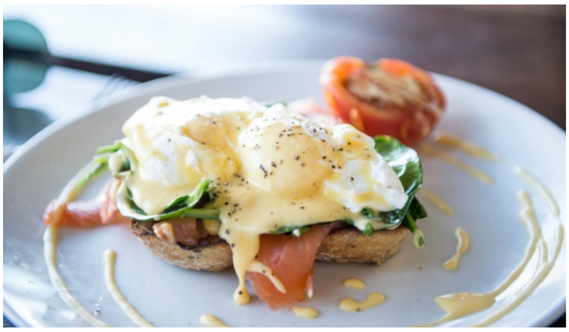

Eggs benedict with smoked salmon on toast

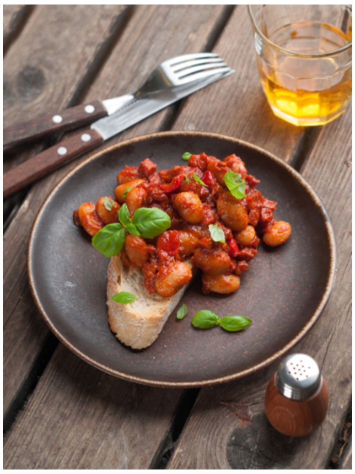

Beans cooked in tomato and onion on toast

19. Looking at products 1 and 2, which one has the most amount of salt?

Pack Size: 425g<br>Serving Size: 69g<br>Servings per packet: 4		
	Per Serve	Per 100g
Energy	327kJ	474kJ
Protein	17.7g	25.7g
Fat, total	0.7g	1.0g
- Saturated	0.3g	0.4g
Carbohydrate, total<br>-Sugars	<1g	<1g
Sodium	311mg	450mg
< means less than		

Canned tuna 1

Pack Size: 185g<br>Serving Size: 60g<br>Servings per packet: 2		
	Per Serve	Per 100g
Energy	284kJ	474kJ
Protein	15.46g	25.7g
Fat, total	0.6g	1.0g
- Saturated	0.2g	0.4g
Carbohydrate, total<br>-Sugars	<1g	<1g
Sodium	210mg	350mg
< means less than		

Canned tuna 2

20. Which of these two products is better for heart health? Circle the best choice.

Nutrition Information<br>Serving Size: 83g<br>Servings per pack: 8		
	Per Serving	Per 100g
Energy	773kJ	931kJ
Protein	9.0g	10.8g
Fat, total	4.0g	4.8g
-saturated	0.5g	0.6g
Carbohydrate	25.0g	30.1g
-sugars	1.6g	1.9g
Dietary Fibre	6.1g	7.3g
Sodium	268mg	323mg

Bread 1

Nutrition Information<br>Serving Size: 60g<br>Servings per pack: 11		
	Per Serving	Per 100g
Energy	612kJ	1020kJ
Protein	5.0g	8.4g
Fat, total	1.2 g	2.0g
-saturated	0.4g	0.6g
Carbohydrate	27.8g	46.31g
-sugars	1.4g	2.4g
Dietary Fibre	1.6g	2.7g
Sodium	240mg	400mg

Bread 2
