# Supplementary material for: Knowledge, Attitudes, and Behaviours Concerning the Mediterranean Diet Among Older Adults in Australia
Source: J Community Health. 2023 Jun 8;48(6):951–62. doi: 10.1007/s10900-023-01237-1 (PMC10248335; doi:10.1007/s10900-023-01237-1)
Supplement: Supplementary file 2 — Supplementary Material 2 [file 10900_2023_1237_MOESM2_ESM.pdf]

21. How would you rate the quality of your overall diet (High quality = high in fresh foods, low in processed foods)?

- ☐ Excellent
- ☐ Very good
- ☐ Good
- ☐ Average
- ☐ Poor

22. The healthiness of foods has little impact on my food choices.

- ☐ Strongly disagree
- ☐ Disagree
- ☐ Somewhat disagree
- ☐ Neither or agree or disagree
- ☐ Somewhat agree
- ☐ Agree
- ☐ Strongly agree

23. It is important for me that my daily diet contains a lot of vitamins and minerals.

- ☐ Strongly disagree
- ☐ Disagree
- ☐ Somewhat disagree
- ☐ Neither or agree or disagree
- ☐ Somewhat agree
- ☐ Agree
- ☐ Strongly agree

24. I eat what I like and do not worry much about the healthiness of foods.

- ☐ Strongly disagree
- ☐ Disagree
- ☐ Somewhat disagree
- ☐ Neither or agree or disagree
- ☐ Somewhat agree
- ☐ Agree
- ☐ Strongly agree

25. Do you proactively look for nutrition information?

- ☐ Yes
- ☐ No

26. How often do you read the nutrition facts label found on packaged foods?

☐ Most of the time
☐ Sometimes
☐ Rarely
☐ Never

27. I prefer to eat home cooked meals rather than eating out or ready-made meals.

☐ Strongly disagree
☐ Disagree
☐ Somewhat disagree
☐ Neither or agree or disagree
☐ Somewhat agree
☐ Agree
☐ Strongly agree

28. I am interested in tasting new food that I haven't tasted before.

☐ Strongly disagree
☐ Disagree
☐ Somewhat disagree
☐ Neither or agree or disagree
☐ Somewhat agree
☐ Agree
☐ Strongly agree

29. What factors or circumstances would make it difficult (harder) to try a new diet? Please select all that apply.

☐ Cost
☐ Lack of time for cooking
☐ Unable to access healthy food
☐ Inconvenience
☐ Unable to access cooking facilities at home
☐ Satisfied with current diet
☐ Lack of knowledge about diet/nutrition
☐ Food taste preferences
☐ Other (please specify): ____

30. What factors or circumstances would facilitate or promote your willingness to try a new diet? Please select all that apply.

☐ Educational information
☐ One on one time with nutritionist/dietitian
☐ Ongoing nutritional support
☐ Access to food items
☐ Access to meal preparation resources e.g. cooking classes, meal plan, recipes
☐ Group meeting (i.e. attended with other participants) facilitated by nutritionist/dietitian
☐ Other (please specify): ____
